# Labile sleep promotes awareness of abstract knowledge in a serial reaction time task

**DOI:** 10.3389/fpsyg.2015.01354

**Published:** 2015-09-07

**Authors:** Roumen Kirov, Vasil Kolev, Rolf Verleger, Juliana Yordanova

**Affiliations:** ^1^Cognitive Psychophysiology, Institute of Neurobiology, Bulgarian Academy of SciencesSofia, Bulgaria; ^2^Department of Neurology, University of LübeckLübeck, Germany; ^3^Institute of Psychology II, University of LübeckLübeck, Germany

**Keywords:** labile sleep, sleep stage transitions, NREM–REM–NREM transitions, explicit knowledge, insight, serial reaction time task

## Abstract

Sleep has been identified as a critical brain state enhancing the probability of gaining insight into covert task regularities. Both non-rapid eye movement (NREM) and rapid eye movement (REM) sleep have been implicated with oﬄine re-activation and reorganization of memories supporting explicit knowledge generation. According to two-stage models of sleep function, oﬄine processing of information during sleep is sequential requiring multiple cycles of NREM and REM sleep stages. However, the role of overnight dynamic sleep macrostructure for insightfulness has not been studied so far. In the present study, we test the hypothesis that the frequency of interactions between NREM and REM sleep stages might be critical for awareness after sleep. For that aim, the rate of sleep stage transitions was evaluated in 53 participants who learned implicitly a serial reaction time task (SRTT) in which a determined sequence was inserted. The amount of explicit knowledge about the sequence was established by verbal recall after a night of sleep following SRTT learning. Polysomnography was recorded in this night and in a control night before and was analyzed to compare the rate of sleep-stage transitions between participants who did or did not gain awareness of task regularity after sleep. Indeed, individual ability of explicit knowledge generation was strongly associated with increased rate of transitions between NREM and REM sleep stages and between light sleep stages and slow wave sleep. However, the rate of NREM–REM transitions specifically predicted the amount of explicit knowledge after sleep in a trait-dependent way. These results demonstrate that enhanced lability of sleep goes along with individual ability of knowledge awareness. Observations suggest that facilitated dynamic interactions between sleep stages, particularly between NREM and REM sleep stages play a role for oﬄine processing which promotes rule extraction and awareness.

## Introduction

Sleep has been identified as a critical brain state involved in consolidation of both explicit and implicit memories (Maquet, 2001; Smith, 2001; Born et al., 2006; Walker and Stickgold, 2006; Diekelmann and Born, 2010), where consolidation refers to a post-learning process that stabilizes and strengthens the new memory traces established at learning (Lechner et al., 1999; Rasch and Born, 2013). Recent studies have shown that sleep may not only stabilize but also reorganize memory representations such that performance after sleep can qualitatively differ from what has been originally encoded (Fenn et al., 2003; Wagner et al., 2004; Ellenbogen et al., 2007). This reorganization has been first prompted in the study of Wagner et al. (2004) where a hidden regularity was implemented in a task to be learned (the number reduction task, NRT). Acquiring explicit knowledge of this hidden regularity (i.e., gaining insight into it) allowed participants to find an alternative direct solution of the task. Wagner et al. (2004) found that sleep enhanced the probability of gaining insight into the covert task structure, as evidenced by a substantially higher number of participants who discovered the hidden regularity (solvers) when the test was performed after sleep compared with wakefulness. Using the same task, Darsaud et al. (2011) have revealed that after sleep, only in participants who gained awareness of the hidden rule, were neural response patterns transformed overnight. Specifically, overnight modulation was observed in the ventral medial prefrontal cortex, a region implicated in the consolidation of memory and uniquely activated before gaining insight at post-sleep retest. Notably, however, already at implicit training before sleep, the neural responses of solvers and non-solvers differed because areas mediating controlled processes (frontal and parietal cortices and the insula) were more active in future solvers, in contrast to significant hippocampal activation in non-solvers (Darsaud et al., 2011). Thus, oﬄine reorganization of encoded memories during sleep is related to subsequent explication of abstract knowledge. Yet, cognitive strategies during encoding may critically determine the oﬄine consolidation supporting subsequent insight.

According to Lewis and Durrant (2011) sleep, in particular the slow-wave sleep (SWS) fraction of non-rapid eye movement (NREM) sleep supports abstraction by re-activating in an overlapping manner memories that are common to several representations. This may lead to strengthening of common elements. In contrast to idiosyncratic elements, these common elements undergo a preferential cortical consolidation either actively (Diekelmann and Born, 2010; Rasch and Born, 2013) or passively (Tononi and Cirelli, 2003, 2006, 2014; Lewis and Durrant, 2011), thus potentiating the oﬄine formation of a new neural representation. Shared features thus undergo selective strengthening and subserve integration, abstraction of rules, insight into hidden solutions, and false memory formation (Lewis and Durrant, 2011). This model is substantiated by findings from both animal and human studies demonstrating that neural patterns of specific behaviors during wake are reactivated during SWS (Wilson and McNaughton, 1994; Lee and Wilson, 2002; Huber et al., 2004, 2006; Rasch et al., 2007; O’Neill et al., 2010).

Previous studies using tasks with hidden regularities have provided evidence for the role of SWS for explicit knowledge generation after sleep. Employing a split-night design, where the role of early night sleep, rich in SWS, and late-night sleep, rich in rapid eye movement (REM) sleep, could be explored separately, Yordanova et al. (2008) have demonstrated that SWS, but not REM sleep, plays a role for the transformation of implicit knowledge generated before sleep to explicit (conscious) knowledge after sleep. The major observation was that the rate of subjects who gained insight into NRT after sleep on the basis of pre-sleep implicit knowledge was significantly higher across early- than late-night sleep. In contrast, late-night subjects preferentially preserved rather than transformed implicit knowledge acquired before sleep (Yordanova et al., 2008). Also, SWS alters the processing of items predicted by the hidden NRT regularity by inducing changes of both information-based processes and functional brain states toward insightful solutions (Yordanova et al., 2009a, 2010). Within the information- and process-based distinction of consolidation, it has been further demonstrated that SWS promotes insight after sleep by consolidating mainly the information that had been encoded and learned explicitly before sleep (Yordanova et al., 2009b), with slow sleep spindles during SWS supporting implicit-to-explicit knowledge transformation (Yordanova et al., 2012). Similarly, a more recent nap study provided further evidence for the enhancing role of SWS for insight solutions (Beijamini et al., 2014).

While these reports emphasize the key role of SWS for explicit knowledge generation, REM sleep also has been implicated with mediating knowledge awareness (Edwards et al., 2013). Walker et al. (2002b) have found that awakenings during REM sleep produce a significant increase in the rate of associative anagram solving relative to awakenings during NREM sleep, suggesting that the neurophysiology of REM sleep maintains cognitive processing that is more flexible than that of NREM sleep. Likewise, REM sleep enhances more the integration of unassociated information for creative problem solving as compared to NREM sleep (Cai et al., 2009). Stickgold et al. (1999) also have pointed to the specific role of REM sleep in associative memory systems, which may be critical for the formation of new abstract representations (Walker and Stickgold, 2010; Stickgold and Walker, 2013). In support, Peigneux et al. (2003) have reported that brain regions involved during learning a serial reaction time task (SRTT), where a hidden regularity was probabilistic, were re-activated during subsequent REM sleep.

Together, these previous studies reveal a role for both SWS and REM sleep in bringing implicitly learned information to awareness. According to the sequential hypothesis (Giuditta et al., 1995), memories acquired during wakefulness are processed during sleep in two serial steps occurring during SWS and REM sleep. Specifically, it is suggested that during SWS, memories are distinguished from irrelevant or competing traces that undergo downgrading or elimination; during REM sleep, retained processed memories are stored again and integrated with preexisting memories (Giuditta, 2014). With regard to “recovery sleep function,” Vyazovskiy and Delogu (2014) also propose that NREM and REM sleep have distinct and complementary contributions to the overall function of sleep. They suggest that functionally interconnected neuronal networks during NREM sleep enable information processing, synaptic plasticity, and prophylactic cellular maintenance (“recovery process”). In turn, periodic excursions into an activated brain state – REM sleep – perform “selection” of recovered brain networks. Targeting specifically the oﬄine functions of sleep to unitize, assimilate, and abstract memory representations, Walker and Stickgold (2010) and Stickgold and Walker (2013) propose that the NREM sleep represents an initial stage of oﬄine processing, during which new episodic memories are preferentially consolidated by keeping their characteristics separate and distinct. By contrast, at a second, REM-dependent stage, these newly encoded and NREM sleep-consolidated memories are integrated into associative networks supporting integration with old memory schemes, rule extraction, and generalization. Critically, with regard to natural sleep architecture, effective integration of these memories is suggested to take several NREM–REM cycles or even multiple nights before optimal representations are complete (Walker and Stickgold, 2010). In the same line, Llewellyn and Hobson (2015) posit a key role for REM after NREM sleep to incorporate emotional information into nodes of mentally translated new episodic memories.

Regarding the dynamic roles of NREM and REM sleep for explicit knowledge generation within a two stage model of sleep function (Walker and Stickgold, 2010), we propose that the interactions between sleep stages might be critical for integrating memories which support awareness after sleep. In the present study, we test the hypothesis that the increased frequency of transitions between sleep stages, in particular between NREM and REM sleep stages, is associated with the ability of explicit knowledge generation after sleep.

## Materials and Methods

### Participants

Fifty-three students at the University of Lübeck (28 female) participated in the study as part of a larger experiment designed to investigate effects of sleep on hemisphere-specific processing. Participants were between 20 and 30 years of age (mean 23.4 ± 2.16 years), had normal or corrected to normal vision as well as normal color vision, were right-handed (evaluated according to the Edinburgh Handedness Inventory, Oldfield, 1971) and did not have histories of neurologic, psychiatric, sleep disturbances, or irregular sleep-wake schedules. Before and during the experiment, no drugs, alcohol, or caffeine drinks were used by the subjects. The experiment was conducted in the sleep electroencephalography (EEG) laboratory at the Department of Neurology at the University of Lübeck. The study was performed according to the clinical standards of the Declaration of Helsinki and was approved by the university’s Ethic Committee. All participants received monetary compensation (60 €) for their participation and gave informed written consent before the study.

### Experiment

Participants performed a version of the serial response time task (Nissen and Bullemer, 1987) where stimuli were presented in the left or right visual half-field (varying across participants, see Verleger et al., 2015, for details) with a first, practice session in the evening and a second, test session in the morning after sleep.

#### Stimuli and Task

The task was a four-choice visual motor task, in which motor responses with four fingers of one hand had to be selected to four colored circles. Colored circles were blue, red, yellow, or green. Their center was located 4.5° laterally from the center of the white screen at horizontal midline. A dark-gray circle of same size was presented at the other side of the screen symmetrically to the color circle. Index to little fingers of the responding hand rested at the four active keys of a custom-made keyboard which contained sets of four keys for either hand. In each trial, a colored circle (blue or red or yellow or green) was presented on one side of the screen, always left of fixation for half of participants (*n* = 28) and always right for the other half (*n* = 25). Responses to the circles had to be made with the ipsilateral hand, by pressing the index, middle, ring, or little finger correspondingly to blue, red, yellow, or green circles, respectively. The stimulus was presented for 200 ms, and the next color circle appeared 800 ms after the correct response.

**FIGURE 1:**
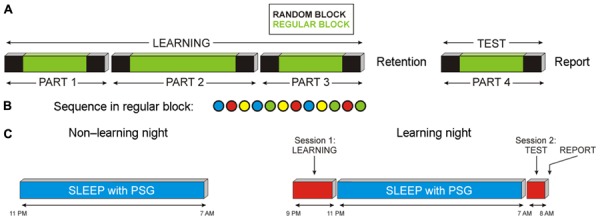
**Schematic presentation of the experimental design. (A)** SRTT was performed in four parts. Unknown to participants, each part was divided in three blocks. Blocks indicated in black contained random sequences of stimuli (random block); blocks indicated in green contained regularly ordered stimuli (regular blocks). Participants practiced SRTT in evening sessions (LEARNING), and performed a TEST session after retention period. **(B)** Illustration of the fixed sequence of stimuli in regular blocks. **(C)** Timing of the experiment in the non-learning and learning nights.

Task structure shown in **Figure 1A** followed the design used by Cohen et al. (2005). From participants’ point of view, the task during learning consisted of three episodes with self-terminated breaks between episodes, where one of the four colors appeared in each trial and had to be responded by pressing the appropriate key. The number of trials in each episode was 280, 400, and 280, altogether 960. Untold to participants, each of the three episodes was a “sandwich” where the outer trials (first 50 and last 50) were random, whereas the inner trials repeated a fixed sequence of 12 stimuli (15, 25, and 15 times in the three episodes). The fixed sequence (**Figure 1B**) was B R Y B G Y R B Y G R G (meaning Blue, Red, Yellow, and Green). During test, the same “sandwich” structure was used, with the outer 50 trials being random and the inner 180 trials following the fixed sequence of 12 elements. Similar to learning, participants were not informed about the occurrence of regular sequences during test. After the testing session, subjects filled in a questionnaire to probe their explicit knowledge related to the hidden task structure as well as possible strategies used during task performance.

#### Procedure

Participants spent an adaptation (non-learning) night in the laboratory with a polysomnographic (PSG) recording, followed after 2–10 days (∼7 days) by the experimental night which was preceded by the learning session and followed by the test session (**Figure 1C**). For all participants, the non-learning night was before the learning night. For the learning night, participants reported to the laboratory at ∼20:00 h. After placement of electrodes for EEG/PSG recording, they performed the task (three blocks of practice) and thereafter went to bed at ∼22:30 h. After 8 h of sleep, participants were awakened at ∼07:00 h. They were only awakened from light sleep stages 1 or 2 to avoid cognitive disturbances that can occur after awakenings from SWS or REM sleep. Finally, participants performed the test session (one block) starting at ∼07:30 h (**Figure 1C**). Subjective levels of sleepiness, activation, boredom, concentration, and motivation were assessed on five-point scales immediately before and after each session of practice (learning) and retest.

### Sleep EEG Recording and PSG Analysis

During the two nights (non-learning and learning), EEG was recorded with Ag–AgCl electrodes (Easycap, http://www.easycap.de) from 26 scalp electrodes according to the International 10/20 system: F7, F3, Fz, F4, F8, FC3, FCz, FC4, T7, C3, Cz, C4, T8, CP5, CP1, CP2, CP6, P7, P3, Pz, P4, P8, PO7, PO8, O1, O2 (BrainAmp MR plus, Gilching, Germany, cut-off frequencies DC and 250 Hz, sampling rate 500/s). Additional electrodes were placed at the nose-tip for reference and at Fpz as a ground. Also, electromyogram (EMG) using submentally attached electrodes, and vertical (from electrodes placed above and below the right eye) and horizontal (from electrodes placed on both outer canthi of the orbits) electrooculogram (EOG) were recorded. Analyzes were performed by means of Brain Vision Analyzer 2.1 (Gilching, Germany) and specially designed software on Matlab R2013b (The MathWorks Inc.).

Off-line PSG analysis including EEG (C3 and C4), EMG, and EOG was performed. PSG data were analyzed visually in 30-s epochs according to standard criteria (Rechtschaffen and Kales, 1968) by two experienced raters blind to participants’ age, gender, and behavioral performance. The distribution of the different sleep stages in the non-learning and learning nights showed normal sleep architecture.

In addition, the number of sleep stage transitions (SST) was measured. This included (1) transitions to wake after sleep onset from Stages 2 (S2) and SWS of NREM sleep, and from REM sleep, and from wake to these sleep stages; (2) transitions from NREM to REM sleep, as well as from REM to NREM sleep (NR–RN); (3) all transitions between Stage 1 (S1), S2, and SWS. The numbers of each type of SSTs were normalized separately by calculating SST per hour of total sleep time.

### Explicit Knowledge Groups

Following the experimental protocol of Nissen and Bullemer (1987), at the end of the test session, participants were asked to report verbally if they had detected any regularity in the appearance of stimuli and, if so, to write on paper any regular sequence they had noted. To quantify the gain of explicit sequence-specific knowledge (ExK) in the SRTT, participants were scored from 1 to 5. In case of no sequence being detected, the participant was scored with 1. Based on their written reproduction, participants were scored with 2 if they could recover a single correct sequence of 3–4 items, with 3 if they reproduced two correct sequences of 3–4 items each, with 4 if they could reproduce a correct sequence of more than 8 items of the 12-item order, and with 5 if they were able to report the whole sequence of 12 items. For example, a participant was scored with 2 if he/she was able to reproduce correctly YBGY or GYR (a correct 3- to 4-item fragment of the 12-item sequence). An example of reporting 2 correct separate fragments is YBG and RBYG (scored 3), in contrast to, e.g., a correct BRYBGYRBY reproduction scored 4. Participants scored with 1 (*n* = 30) and 2 (*n* = 11) were assigned to the group of no gain of ExK about sequence (No-ExK, non-solvers, *n* = 41), those scored with 3 (*n* = 6), 4 (*n* = 4), and 5 (*n* = 2) were assigned to the group of gain of ExK about sequence after sleep (ExK, solvers, *n* = 12).

### Statistical Analysis

All PSG parameters including the normalized SST were analyzed using a mixed analysis of variance (ANOVA) design with the between-subjects variable Group (no-ExK vs. ExK, i.e., non-solvers vs. solvers) and the within-subjects variable night (non-learning vs. learning). In addition, Pearson’s two-tailed correlations and a multiple regression analysis (step-wise model) were conducted as detailed in the Section “Results”.

## Results

Group mean values of the analyzed PSG parameters are presented in **Table 1** and statistical results from ANOVAs are presented in **Table 2**. The tables demonstrate that none of the major PSG parameters (total time in bed, total sleep time, sleep onset latency, sleep efficiency, latencies to SWS and REM sleep, and duration of all sleep stages) differed between the groups of solvers and non-solvers [Group, *F*(1/51) < 2.2, *p* > 0.15], nor were between-group differences in these PSG parameters modulated significantly by Night [Group × Night, *F*(1/51) < 3.9, *p* > 0.05]. While total time in bed and total sleep time did not differ between the learning and non-learning nights, sleep onset latency, latencies to SWS and REM sleep and the amount of wake, Stage 1, Stage 2 of NREM sleep and of movement time decreased significantly in the learning relative to the non-learning night, showing the effect of becoming adapted to the lab environment. Correspondingly, sleep efficiency and the amounts of SWS and REM sleep significantly increased (*p* < 0.001; **Table 2**).

**Table 1:** Sleep PSG parameters for the non-learning and learning night.

	No explicit knowledge (*n* = 41)		Explicit knowledge (*n* = 12)	
	Non-learning night	Learning night	Non-learning night	Learning night
**Duration measures (min)**
Total time in bed (TIB)	495 ± 69	477 ± 53	481 ± 51	461 ± 48
Total sleep time (TST)	469 ± 39	464 ± 45	452 ± 36	448 ± 46
Sleep onset latency	25 ± 10	13 ± 10	29 ± 13	13 ± 6
SE (TST/TIB) %	95 ± 2	97 ± 2	94 ± 3	97 ± 2
Latency to SWS	25 ± 15	13 ± 4	34 ± 29	15 ± 6
Latency to REM sleep	85 ± 36	60 ± 19	83 ± 33	56 ± 16
**Duration of sleep stages (% of TST)**
Wake	4.6 ± 3.9	2.2 ± 4.6	4.8 ± 4.2	0.8 ± 1.2
Stage 1	4.5 ± 2.1	1.9 ± 1.8	5.4 ± 2.1	1.2 ± 0.6
Stage 2	49.1 ± 6.4	44.5 ± 6.5	47.8 ± 6.4	44.8 ± 6.8
SWS (Stages 3 + 4)	14.9 ± 3.6	20.3 ± 5.2	15.1 ± 4.1	21.5 ± 4.5
REM sleep	24.9 ± 4.7	29.7 ± 6.1	25.3 ± 5.1	30.5 ± 4.7
Movement time	1.9 ± 1.2	1.9 ± 1.8	1.8 ± 0.9	1.3 ± 0.9
**Rate of SST (Number of transitions per hour)**
Total SST	4.5 ± 0.9	4.1 ± 0.8	6.0 ± 1.1	5.9 ± 1.1
SST to Wake	1.2 ± 0.5	0.5 ± 0.4	1.0 ± 0.5	0.4 ± 0.4
SST NR–RN	1.4 ± 0.4	1.5 ± 0.3	2.1 ± 0.4	2.1 ± 0.3
SST: S1, S2, and SWS	1.3 ± 1.1	2.0 ± 0.9	2.6 ± 1.0	3.3 ± 1.0

*Shown are group mean values ± SD. SE, sleep efficiency; SWS, slow wave sleep; REM, rapid eye movement; SST, sleep stage transitions; NR, non-REM (NREM) to REM (transitions); RN, REM to NREM (transitions); SST: S1, S2 and SWS, transitions between Stage 1, Stage 2 of NREM sleep and SWS*.

**Table 2:** ANOVA results.

	Main effects				Interaction	
	Group		Night		Group × Night	
	*F*	*P*	*F*	*p*	*F*	*p*
TIB	2.1	0.2	2.7	0.1	0.2	0.9
TST	2.2	0.1	0.4	0.5	0.1	0.9
SOL	0.3	0.7	63.6	<0.001	1.3	0.3
SE (TST/TIB) %	0.3	0.6	56.3	<0.001	0.9	0.3
**Latencies to sleep stages**						
SWS	3.1	0.1	24.9	<0.001	1.1	0.3
REM sleep	0.2	0.7	18.2	<0.001	0.3	0.7
**Duration of sleep stages (% of TST)**						
Wake	0.0	0.9	14.8	<0.001	0.1	0.4
Stage 1	1.4	0.3	76.2	<0.001	4.0	0.1
Stage 2	0.1	0.8	11.5	0.001	0.6	0.5
SWS (Stages 3+4)	0.4	0.5	42.4	<0.001	0.3	0.6
REM sleep	0.2	0.7	32.2	<0.001	0.2	0.7
Movement time	0.6	0.4	4.7	0.04	0.2	0.9
**Rate of SST**						
Total SST	**37.2**	**<0.001**	10.2	0.002	**4.0**	**0.05**
SST to Wake	1.7	0.2	74.3	<0.001	0.2	0.7
SST NR–RN	**37.2**	**<0.001**	5.2	0.03	1.2	0.3
SST: S1, S2, and SWS	**18.5**	**<0.001**	19.4	<0.001	0.0	0.9

*Group: no explicit knowledge (*n* = 41) vs. explicit knowledge (*n* = 12); Night: non-learning night vs. learning night. F, Fisher coefficient; *p*, level of significance. Degrees of freedom are 1.51 for all effects. Significant effects including group are in bold. TIB, total time in bed; TST, total sleep time; SOL, sleep onset latency; SE, sleep efficiency; SWS, slow wave sleep; REM, rapid eye movements; SST, sleep stage transitions; NR, non-REM (NREM) to REM sleep (transitions); RN, REM to NREM sleep (transitions); SST: S1, S2 and SWS, transitions between Stage 1, Stage 2 of NREM sleep and SWS*.

**FIGURE 2:**
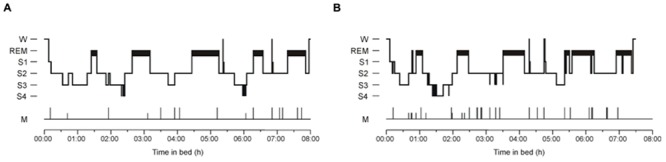
**Representative hypnograms of **(A)** a participant who did not gain explicit knowledge and **(B)** a participant who gained explicit knowledge**. W, Wake; S1, Stage 1; REM, rapid eye movement sleep; S2, Stage 2 non-REM sleep; S3, Stage 3 of slow wave sleep (SWS); S4, Stage 4 of SWS; M, epochs containing movements.

**Figure 2** illustrates hypnograms of two representative participants with and without explicit knowledge after sleep. The total rate of transitions was larger in solvers than non-solvers (Group, *p* < 0.001) and was reduced during post-learning sleep (Night, *p* = 0.002), but this decrease was less expressed in solvers than in non-solvers (Group × Night, *p* = 0.05; **Table 1**). These effects were not due to transitions from sleep stages to wake after sleep onset which did not differ between solvers and non-solvers (Group, *p* = 0.2). Although the rate of transitions to wake was significantly reduced in the learning relative to the first non-learning night (Night, *p* < 0.001), this effect was not modulated by the ability of explicit knowledge generation after sleep (Group × Night, *p* = 0.7).

**FIGURE 3:**
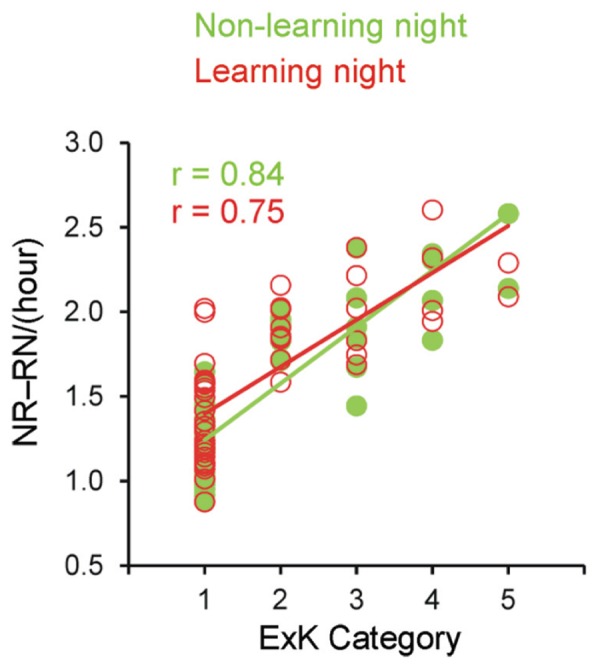
**Correlations between the explicit knowledge category and transitions from non-rapid eye movement (NREM) sleep to REM sleep and from REM to NREM sleep on both the non-learning and learning nights**. ExK, explicit knowledge; NR, rate of transitions from NREM to REM sleep per hour of total sleep time; RN, rate of transitions from REM to NREM sleep per hour of total sleep time.

Rather, as **Tables 1** and **2** and **Figure 3** show, these effects on total rate of transmission were due to transitions between sleep stages. First, the rate of NR–RN transitions was significantly larger in solvers than non-solvers (Group, *p* < 0.001), with this difference being independent from the post-learning enhancement of NR–RN transitions (Night, *p* = 0.03; Group × Night, *p* = 0.3). Second, transitions between sleep stages other than NR–RN (Stage 1 and Stage 2 of NREM sleep and SWS) was significantly higher in solvers than non-solvers in the two nights (Group, *p* < 0.001; Group × Night, *p* = 0.9). Pre-sleep learning was associated with a higher rate of SST between light sleep stages (Stage 1 and Stage 2 of NREM sleep) and SWS (Night, *p* < 0.001).

These ANOVA results were mirrored in results of correlating the ExK scoring from 1 to 5 with each of these parameters for either night separately (non-learning and learning): total rate of transitions (*r* = 0.78 and 0.81, *p* < 0.001); rate of transitions NR–RN (*r* = 0.84 and 0.75, *p* < 0.001); rate of transitions between other sleep stages (*r* = 0.44 and 0.66, *p* < 0.001). The correction for multiple tests (two nights × three parameters) yielded *p* = 0.006, validating the significance of the so obtained correlations. In a multiple regression stepwise analysis, these transition parameters, age (in months) and gender were included as predictors of ExK scores (1–5). **Figure 3** displays these correlations for the NR–RN transitions. A significant model solution [*r* = 0.837, *r*^2^ = 0.701; *F*(1/51) = 119.4, *p* < 0.001] extracted only the rate of NR–RN as a predictor of the amount of explicit knowledge generation after sleep (*B* = 2.1, β = 0.837, *t* = 10.9, *p* < 0.001), with none of the other variables being selected as a predictor.

## Discussion

Based on models about the integrative function of sleep stages for oﬄine information processing (e.g., Giuditta et al., 1995; Walker and Stickgold, 2010; Stickgold and Walker, 2013; Giuditta, 2014; Llewellyn and Hobson, 2015), the present study explored the association between the frequency of transitions between sleep stages and ability to generate explicit knowledge after sleep. The SRTT was used (in a lateralized version) to induce implicit encoding of a hidden sequence before sleep. The amount of explicit knowledge about sequence-specific information was estimated after sleep and correlated with the rate of SST during a full-night sleep.

According to the major results, gain of explicit knowledge about task regularity following incidental pre-sleep learning of SRTT was strongly associated with increased rate of transitions between NREM and REM stages of sleep. Additionally, this explicit knowledge was related to an overall increase in SST, including also those between sleep Stage 1, Stage 2 of NREM sleep, and SWS. However, the increased rate of NR–RN transitions specifically predicted the amount of explicit knowledge after sleep as indexed by the multiple regression model. Together, these results reveal that in individuals capable of explicating abstract information, there is enhanced lability of sleep stages marked by facilitated dynamic transitions between them, particularly between NREM and REM sleep stages.

Sleep stage shifts have been identified as a reliable marker of sleep continuity, in addition to standard measures (e.g., arousal index; Stepanski et al., 1984; Haba-Rubio et al., 2004). In a study of more than 5600 participants, only transitions from sleep stages to awakenings after sleep onset have been demonstrated to affect daily functioning, thus being recognized as markers of fragmented sleep or markers of impaired sleep micro-architecture (Laffan et al., 2010). In contrast, unstable and transitory sleep stages do not index impairment of sleep architecture and are not accompanied by a less restorative function of sleep (Swarnkar et al., 2009; Laffan et al., 2010). Specifically, transitions between NREM and REM sleep stages had no significant effect on self-reported sleep quality and daytime neurobehavioral functions (Laffan et al., 2010). The results of the present study demonstrate that the rate of transitions from sleep stages to wake did not differ between participants generating and not generating explicit knowledge after sleep. If anything, trends toward reduced rate of transitions to wake and a decreased amount of wakes after sleep on set also were observed in participants with explicit knowledge, in addition to preserved indices of sleep efficiency (**Table 1**). Therefore, the increased rate of SST observed here does not reflect fragmented sleep macro-architecture in explicit solvers. Rather, it reveals a specific quality of sleep architecture in relation to the capacity to generate explicit abstract knowledge about implicitly learned hidden regularity.

The observation that the transitions between all sleep stages were significantly more frequent in subsequent ExK solvers indicates that the pronounced facilitation of shifts between REM and NREM episodes is rather an expression of a more fundamental feature of sleep architecture characterized by general lability. This notion is supported by the result that solvers manifested increased rate of transitions in both the non-learning and learning nights. Hence, facilitated inter-stage dynamics during sleep appears as an individual trait characteristic potentiating a predisposition to explicit abstraction. The novel finding here is that such individual traits are marked by features of sleep continuity.

These observations are consistent and extend reports according to which individual ability for post-sleep explicit extraction of regularity may be associated with an increase in other neurophysiologic signature of sleep, namely slow spindles (8–12 Hz) during SWS (Yordanova et al., 2012). In that previous study that used the NRT, data from control nights were not available for analysis, so we could not decide whether this feature was specific to the night after the first task session or reflected some general trait of task solvers. Analyses of sleep spindles from the present SRTT suggest that both alternatives apply (Yordanova et al., submitted). The present observations are also broadly in line with previously established correlations between individual intellectual capacity and Stage 2 of NREM sleep-specific EEG (sleep spindles) signatures (Bódizs et al., 2005; Schabus et al., 2006; Fogel and Smith, 2011). Confirming previous studies with NRT (Wagner et al., 2004; Yordanova et al., 2008, for the relevant early-night group), standard evaluation of PSG parameters including the amount of sleep stages did not capture individual differences between subjects who had the ability to bring knowledge to awareness and those who did not. Thus, labile sleep and facilitated transitions between distinct sleep stages are originally revealed here as a marker for individual capacity of extracting abstract information.

On the other hand, the observation that sleep-stage transitions (excluding those to and from wake) increase in the learning relative to the non-learning night in all participants, independently of the ability to generate explicit knowledge after sleep, indicates that sleep continuity and macro-architecture are sensitive to pre-sleep learning. The experimental setup of the present study [e.g., combined application of regular and random blocks (Cohen et al., 2005), a lateralized version of the classical SRTT (Schmitz et al., 2013; cf. Verleger et al., 2015, for details)], duration of implicit learning sessions of about (30 min, etc.) may have affected specific sleep characteristics (Al-Sharman and Siengsukon, 2014). For instance, a specific increase in both SWS and REM sleep in response to other types of preceding implicit visuomotor learning is well documented (e.g., Maquet, 2001; Tononi and Cirelli, 2014). Our current observations of shortened latencies to SWS and REM sleep and increase in their amounts on the second relative to the first night are in line with the above mentioned effects of pre-sleep learning. Yet, these effects were accompanied by shortened sleep onset latency, improved sleep efficiency and reduced amounts of wake and movement time after sleep onset, consistent with expected influences of adaptation night on sleep (Agnew et al., 1966). Particularly with regard to SST, the decreased rate of total SST and rate of SST to wake also may reflect differences not related to pre-sleep learning in the second night but to the impact of adaptation during the first non-learning night (Agnew et al., 1966). It is a limitation of the present study that the non-learning night served as adaptation night, and that the non-learning and learning nights were not counterbalanced across subjects, nor was an additional purely adaptation night used for control. However, the observed differences in sleep between the two nights may not be readily attributed to adaptation, since in our study, the two nights of sleep (without and with learning) were not consecutive, but were divided by an interval of about 7 days. More important in the context of SST was the observation that the rate of transitions between sleep stages (NR–RN and other, **Tables 1** and **2**) increased after learning, which may not be predicted by effects of adaptation.

The new evidence provided by the current study is that pre-sleep sensorimotor learning of structured information is specifically associated with increased probability of transitions between sleep stages. There are experimental grounds to interpret this result as reflecting the consolidation functions of sleep since post-sleep improvement of both procedural and declarative memories have been linked with Stage 1 (van Dongen et al., 2011), Stage 2 (Walker et al., 2002a; van Dongen et al., 2011; Llewellyn and Hobson, 2015) and SWS stages of NREM sleep (revs. Diekelmann and Born, 2010; Rasch and Born, 2013; Stickgold and Walker, 2013). There can be, however, also a non-consolidation explanation for the increased rates of transitions after learning. Previously, dynamic features of brain electrophysiological states have been exclusively characterized with respect to spatio-temporally identified functional microstates (Lehmann et al., 2009; Lehmann and Michel, 2011). Functional microstates have been shown to operate on different time scales (from hundreds of milliseconds to 16 s, Van De Ville et al., 2010) and to be present in sleep stages (Wehrle et al., 2007; Brodbeck et al., 2012). Functionally, EEG microstates are understood to represent spontaneous fluctuations of activity in large scale brain networks (Koenig et al., 2002; Michel et al., 2009; Britz et al., 2010). They have been discussed as correlates of information processing steps, in the sense of “atoms of thought” (Lehmann and Michel, 2011) tentatively inducing specific spontaneous mentations (Lehmann et al., 1998). The dynamics of this spatio-temporal micro-architecture also has been suggested to drive transitions to sleep stages (Brodbeck et al., 2012). Although in the present study, state dynamics was examined for classical sleep stages, facilitated transitions after pre-sleep learning as a state-dependent feature, and in subjects with high capacity for explicit knowledge abstraction as a trait-dependent feature, may be a reflection of a more global regulation of dynamic brain states associated with neural network functioning.

Notably, the present results extract a specific role of transitions between NREM and REM sleep stages for explicit knowledge generation after sleep. This role is emphasized not only by significant differences between subsequent explicit knowledge vs. no-knowledge, but mainly by the predictive effect of RN–NR transitions on gradual amounts of explicit knowledge recovery. These observations generally substantiate models of sleep function according to which oﬄine processing of memory representations requires the sequential or integrative contributions of both NREM and REM sleep stages (Giuditta et al., 1995; Giuditta, 2014), especially with regard to rule extraction and generalization (Walker and Stickgold, 2010; Stickgold and Walker, 2013). Specifically, pre-sleep learning was found here to increase NR–RN transitions in both solvers and non-solvers pointing to the potentiating effect of pre-sleep encoding and/or activation on the frequency of subsequent NREM–REM interactions. On the other hand, causality between increased rate of RN–NR transitions and explicit knowledge after sleep may not be inferred since higher frequency of NR–RN transitions did not generate explicit rule extraction in non-solvers. Hence, increased rate of NR–RN shifts both after learning and in relation to individual ability for knowledge explication can be accounted for by the assumption that multiple NREM–REM cycles are required to achieve optimal representations for rule extraction (Walker and Stickgold, 2010). Within this notion, the present results suggest that a critical threshold of transitions rate is needed to reach effective integration of representations, which can only be achieved by individuals manifesting a high background rate of transitions (solvers). The methodology of the present study does not allow specifying exactly which mechanisms are involved in NREM–REM sleep interactions so that knowledge consolidation and abstraction can be enhanced. It can be, however, concluded that such mechanisms of inter-stage interactions are sensitive to information encoded before sleep, in addition to their neuroplasticity modulations.

Theoretical implications of these interactions refer essentially to consecutive iterations of memory consolidation processes supported by multiple transitions between NREM and REM sleep stages (Rasch and Born, 2013; Stickgold and Walker, 2013; Llewellyn and Hobson, 2015), or between other sleep stages, Stage 1 and Stage 2 of NREM sleep (Walker et al., 2002a; van Dongen et al., 2011). Other intriguing perspectives relevant for future studies also exist. One such perspective is raised by the beneficial role of dreaming and dream contents for insightful behaviors (Edwards et al., 2013). Wamsley et al. (2010) have demonstrated that improved task performance at retest after NREM sleep was strongly associated with task-related dream imagery, suggesting that dream experiences reflect the process of oﬄine reactivation of recently formed task memories. Also, wakefulness and dream mentations appear to rely on identical neurophysiologic substrates at macro- and meso-level of organization (Marzano et al., 2011; De Gennaro et al., 2012; Scarpelli et al., 2015). It is plausible that by virtue of the ideomotor potential of mental images (Hommel et al., 2001; Hommel, 2009) enhanced dream recall during wake triggers (or potentiates) the activation of the task-related neural substrate which has been reorganized during sleep (Wamsley et al., 2010), thus promoting access to awareness of previously un-explicated information. As an additional experimental direction, investigations of oﬄine emotional processing can be considered. In fact, an enhancing function of dream content for insight may be grossly substantiated by powerful emotional activations integrated in the consolidated memory items during REM sleep (Nishida et al., 2009; Goldstein and Walker, 2014), thus rendering them more distinct (Llewellyn, 2013; Llewellyn and Hobson, 2015). Multiple iterations of these processes may optimize the node structure of integrative gist, promoting knowledge extraction and awareness.

## Author Contributions

Substantial contributions to the conception and design of the work: RK, RV, JY; Acquisition, analysis of data: RK, VK, RV, JY; Interpretation of data: RK, VK, RV, JY; Drafting the work and revising it critically for important intellectual content: RK, VK, RV, JY; Drafting the work and revising it critically for important intellectual content: RK, VK, RV, JY; Final approval of the version to be published: RK, VK, RV, JY. Agreement to be accountable for all aspects of the work in ensuring that questions related to the accuracy or integrity of any part of the work are appropriately investigated and resolved: RK, VK, RV, JY.

## Conflict of Interest Statement

The authors declare that the research was conducted in the absence of any commercial or financial relationships that could be construed as a potential conflict of interest.
